# Uncovering genetic variations in HTR2A and miRNAs drivers of breast cancer risk in African American women

**DOI:** 10.46439/breastcancer.6.037

**Published:** 2026

**Authors:** Nicole Retland, Karl M. Thompson, Joseph I. Aubee, Victor Apprey, Somiranjan Ghosh, Robert Copeland, Yasmine Kanaan, Yayin Fang, Ali Ramadan, Hassan Brim, Muneer M. Abbas

**Affiliations:** 1 Fielding Graduate University, Center for the Advancement of STEM Leadership, Santa Barbara, CA, USA; 2 Howard University, Department of Microbiology, Washington, DC, USA; 3 The National Human Genome Center, Howard University, Washington, DC, USA; 4 Howard University, College of Arts and Sciences, Washington, DC, USA; 5 Howard University, Department of Pharmacology, Washington, DC, USA; 6 Howard University, Department of Biochemistry, Washington, DC, USA; 7 Howard University, Department of Pathology, Washington, DC, USA

**Keywords:** Triple-Negative breast cancer, African American women, HTR2A gene, Single nucleotide polymorphisms, microRNA, Gene regulation, Breast cancer disparities, Precision medicine

## Abstract

**Background/Objectives:**

Triple-negative breast cancer (TNBC) disproportionately affects African American (AA) women, yet the molecular mechanisms remain poorly understood. Emerging evidence suggests that microRNAs (miRNAs) may play a critical role in cancer progression. This study investigated the association of HTR2A genetic variants and miRNA dysregulation in AA women with breast cancer.

**Methods:**

A total of 417 AA women (192 with breast cancer, 225 controls) were genotyped for selected HTR2A SNPs. Functional predictions and molecular modeling assessed structural impact. TNBC cell lines were treated with an HTR2A agonist, and miRNA expression was profiled using the NanoString, and bioinformatic tools were applied to identify miRNA targets and associated oncogenic pathways.

**Results:**

Three SNPs (rs6304, rs6311, and rs1745837) were significantly associated with breast cancer risk (e.g., rs6311 dominant model: OR = 11.98, 95% CI: 6.63–21.66, p < 0.0001). The rs6304 demonstrated predicted protein destabilization. Six oncogenic miRNAs (including miR-92a-3p and miR-377–3p) were significantly upregulated (p < 0.05) and involved in PI3K/AKT, JAK/STAT, and IL-6 pathways.

**Conclusions:**

HTR2A genetic variation, miRNA dysregulation, and increased breast cancer risk in AA women. These findings suggest that HTR2A variants and miRNA profiles may serve as potential biomarkers for risk stratification and targeted therapeutic approaches in TNBC.

**Simple Summary:**

African American women face a higher risk of developing a particularly aggressive type of breast cancer called TNBC. This study explores how changes in a specific serotonin receptor gene, known as HTR2A, might contribute to this risk. By analyzing the DNA of AA women and conducting lab experiments, the researchers identified genetic variations that are more common in women with breast cancer. They also discovered that these genetic changes may affect the way small molecules called microRNAs control cell growth. Understanding these links may help scientists find better ways to detect and treat breast cancer in AA women, paving the way for more personalized and effective medical care.

## Introduction

Since its initial discovery in 1948 by the Research Division at the Cleveland Clinic Foundation as a vasoconstrictor substance in serum, 5-hydroxytryptamine (5-HTA), commonly known as serotonin, has undergone extensive investigation within the context of the central nervous system (CNS) [1,2]. Serotonin, a monoamine neurotransmitter, exerts its effects through a complex interplay with 15 different receptors [3–5] and plays a pivotal role in regulating various physiological processes, including mood, appetite, sleep, and cognitive function within the CNS [6].

Although the role of serotonin in the CNS is well-defined, it is crucial to acknowledge that the majority of serotonin is produced in the periphery by enterochromaffin cells of the gastrointestinal (GI) tract [7,8]. Serotonin produced in the GI tract is stored by platelets and released upon activation [9,10]. Moreover, a multitude of serotonin receptors are expressed in diverse peripheral organs [11,12]. This peripheral serotonin participates in the regulation of various physiological processes, including homeostasis, heart rate, vascular tone, intestinal motility, and cell growth in organs such as the liver, bone, pulmonary arteries, heart, brain, mammary gland, and immune functions [13], and improper balance of peripheral 5-HT has been associated with several diseases [14,15]. Notably, serotonin imbalance has been implicated in cancer progression through its interaction with receptors that are linked to diverse signaling pathways [16–18]. In vitro studies involving MCF-7 cells have demonstrated that serotonin promotes breast cancer cell growth through receptor-mediated pathways [19–21] and confirmed the presence of HTR2A, a G protein-coupled serotonin receptor, on the surfaces of breast cancer (BCa) cells and its mitogenic effects. Another study examining nearly 2,000 BCa samples concluded that 5-HT production and alterations in 5-HT receptor expression increased tumor cell viability, leading to a poor prognosis among women [22].

BCa is a multifaceted disease estimated to affect approximately 32% of women in the United States [5]. Clinically, BCa is often categorized into four subtypes: luminal A (HR+, HER2−), luminal B (HR+, PR+, HER2+), HER2-enriched (ER−, PR−, HER2+), and triple-negative (HR−, PR−, HER2−). However, TNBC, while not a distinct molecular subtype, represents the most aggressive form and disproportionately affects AA women [23,24].

Single-nucleotide polymorphisms (SNPs) have garnered significant attention in breast cancer research due to their potential role in influencing susceptibility and prognosis [25]. These common genetic variations involve the substitution of a single nucleotide base and are widely distributed throughout the human genome [26]. Specific SNPs have been associated with an increased risk of developing breast cancer, impacting gene functions critical for tumor initiation, growth, and metastasis [27].

Understanding the relationship between SNPs and breast cancer can provide valuable insights into the genetic factors contributing to this complex disease, aiding in the development of personalized treatment strategies and risk assessment for individuals at greater risk of breast cancer [28]. Epigenetics, in contrast to SNPs, does not involve altering the underlying deoxyribonucleic acid (DNA) sequence. Instead, gene expression is modified through various cellular mechanisms, including histone modifications, DNA methylation, and non-coding ribonucleic acids (RNA) [29].

microRNA (miRNA) is a small non-coding RNA molecule, approximately 22 nucleotides in length, that can silence gene transcription or induce mRNA degradation through complementary base pairing with mRNA [30–32]. Due to its regulatory function, miRNA has been implicated in numerous diseases, including cancer [33,34]. The progression of BCa can be influenced by the accumulation of genetic and epigenetic factors, which ultimately impact prognosis and treatment.

Despite known disparities in TNBC incidence and outcomes among AA women, the contribution of HTR2A genetic variation and its interaction with miRNA-mediated regulation remains poorly understood. This study aims to address this gap by evaluating the association between HTR2A SNPs and breast cancer risk, and by investigating how these variants may influence miRNA expression and oncogenic signaling pathways.

## Materials and Methods

### Participant recruitment and ethical compliance

This study adhered to the ethical principles of the Declaration of Helsinki, with Institutional Review Board (IRB) approvals under protocols IRB-19–01, NIH #R01 55772, UL1TR000101, and KL2TR000102. Participants were recruited from two NIH-funded studies, “Clinical and Genetic Screening of High-Risk AA BCa Families” and “BCa in Black Women: Gene and Environment Interactions,” as well as the “Breast Cancer Determinants in Women from Washington, DC” cohort. The final cohort (Table 1) comprised 417 AA women, including 192 breast cancer patients and 225 controls, who provided written informed consent, supervised by independent researchers to mitigate conflicts of interest. Controls were frequency-matched to breast cancer patients based on age (**±**2 years), and demographic data, including lifestyle factors, were collected to account for potential confounders. In addition to age-matching, we minimized potential confounding by collecting clinical and demographic information, including self-reported ancestry and lifestyle factors. Variables including BMI, smoking status, and alcohol consumption were recorded but were not included as covariates in regression models due to sample size limitations. While these were not adjusted in the current analysis, their potential influence is acknowledged as a limitation.

### DNA isolation

Blood samples (10 mL) were collected in EDTA tubes and processed within 2 hours to preserve nucleic acid integrity. Genomic DNA was extracted using the Qiagen DNeasy Blood & Tissue Kit, with quality verified by NanoDrop spectrophotometry. All biospecimens were de-identified, and data were stored in encrypted, password-protected databases, adhering to HIPAA guidelines. To minimize selection bias, controls were recruited from the same geographic area as the patients. An independent Data and Safety Monitoring Board (DSMB) provided regular oversight, ensuring protocol compliance and participant safety. De-identified data will be made publicly accessible in repositories such as dbGaP, contingent on IRB approval, to enhance transparency and facilitate future research on genetic risk factors in AA populations.

### *In silico* analyses and genotyping

#### Population-based genetic variation analysis

Data on the human HTR2A gene were retrieved from the National Center for Biotechnology Information (NCBI) database. SNPs were identified from the 1000 Genomes Project Phase 3 dataset by comparing minor allele frequencies (MAFs) between African (AFR) and European (EUR) superpopulations. The AFR superpopulation (n = 661) included individuals from seven subpopulations: ACB, ASW, ESN, GWD, LWK, MSL, and YRI; the EUR superpopulation (n = 503) included CEU, FIN, GBR, IBS, and TSI (Table 2). SNP allele frequency data were obtained from: https://www.ncbi.nlm.nih. gov/variation/tools/1000genomes/?assm=GCF_000001405.25. All variants were aligned to the GRCh38.p7 genome assembly and reported on the forward strand.

#### Genetic variants selection, prediction, and genotyping

Four SNPs (rs6304, rs6311, rs1745837, and rs7322347) (Table 3) were selected based on significant differences in minor allele frequency (MAF) between African (AFR) and European (EUR) populations, identified through Chi-square analysis (p < 0.05). Selection criteria included high Combined Annotation Dependent Depletion (CADD) scores [36] and prior associations with diseases excluding neurological or psychiatric conditions. Genomic locations were confirmed using the UCSC Genome Browser, and functional predictions were performed using the SNPinfo Web Server (NIEHS) [35].

**Figure 1.**
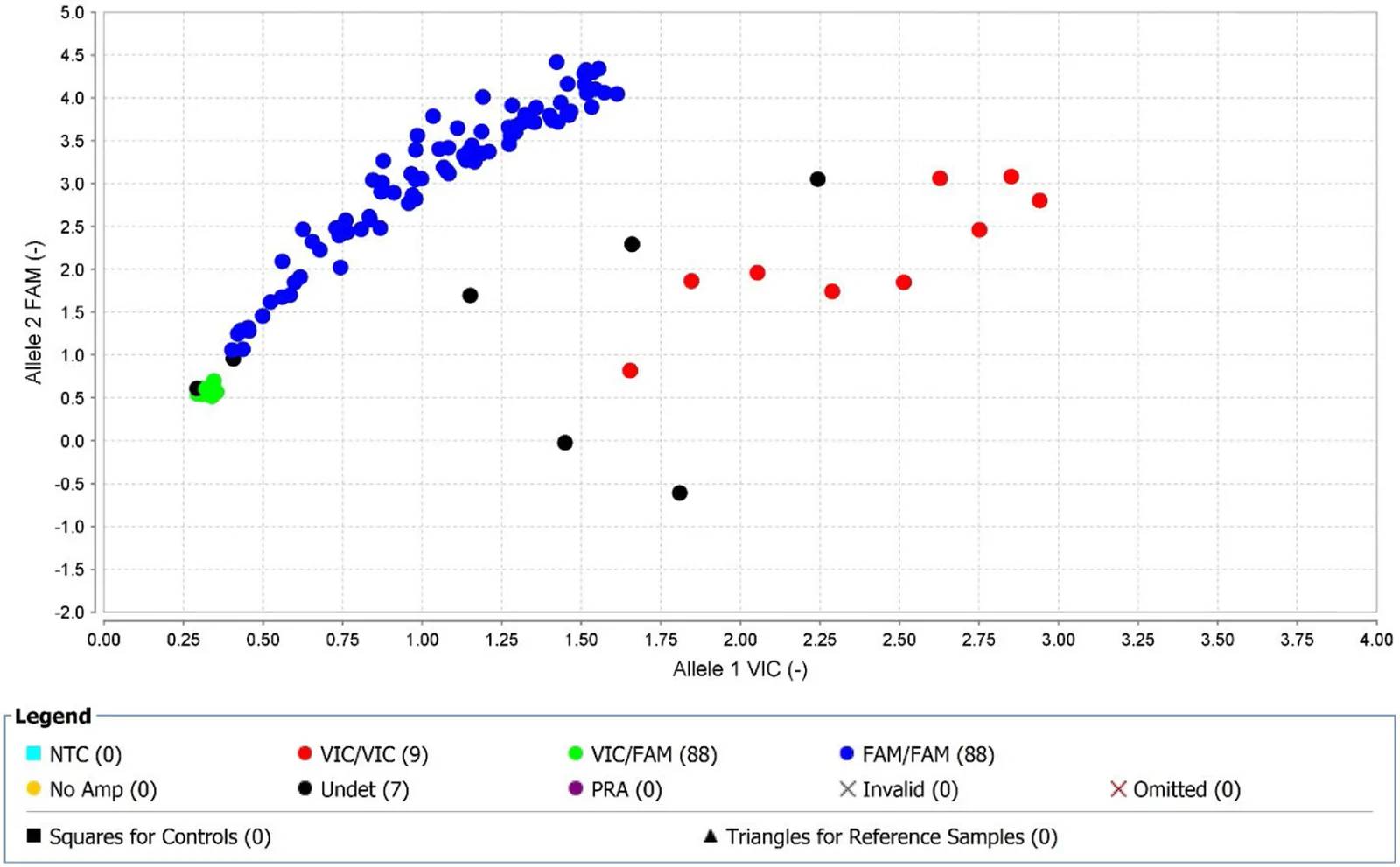
Genotyping results for HTR2A SNP rs6304 using TaqMan allelic discrimination assay. Scatter plot of fluorescence intensities showing Allele 1 (VIC) versus Allele 2 (FAM) signals. Each data point represents an individual sample. Distinct clustering of points indicates three genotype groups (TT, CT, and CC), demonstrating accurate and reproducible SNP genotyping.

Genomic DNA was quantified using Nanodrop 2000. Samples with 260/208 ratios between 1.6 and 1.8 were normalized to 50 ng/μl in hydration buffer. Genotyping was conducted using the TaqMan SNP assay on the 7900HT Fast Real-Time PCR System with a 384-well block. Following the dry method protocol, DNA was added to wells, dried, and combined with master mix, primers, and nuclease-free water to a final volume of 5 μL. Allelic discrimination was performed per Applied Biosystems protocols, and data were analyzed using SDS v2.3 and TaqMan Genotyper Software. Genotyping quality and allele discrimination were confirmed by clear clustering of fluorescence signals, as illustrated in Figure 1.

Scatter plot of fluorescence intensities showing Allele 1 (VIC) versus Allele 2 (FAM) signals. Each data point represents an individual sample. Distinct clustering of points indicates three genotype groups (TT, CT, and CC), demonstrating accurate and reproducible SNP genotyping.

#### Molecular modeling

##### Hardware and software:

Molecular Operating Environment (MOE) is a drug discovery software platform that integrates visualization, modeling, and simulations, as well as methodology development, in one package. For molecular modeling, the computer provided was furnished with Microsoft Windows 7 Professional OS and an Intel Xeon 3.40 GHz dual processor with 64.0 GB of physical memory. All molecular modeling was performed with MOE (Molecular Operating Environment, Chemical Computing Group, Inc.).

##### Data setting:

The three-dimensional (3-D) structure for HTR2A was taken from the RCSB Protein Data Bank (6A93) and imported into the MOE window. Once in MOE, the amino acid sequence was edited to demonstrate the effect of the nsSNP rs6304.

##### Protein stability evaluation:

Dynamut, a web server, analyzes protein dynamics or the effect of single-base mutations on protein stability[35]. For this study, Dynamut was employed to evaluate the effect of the amino acid substitutions encoded by nsSNPs on protein stability. As such, numerical and visualization data were obtained for single-base mutations. To obtain these data fields, relating to PDB ID, mutation, and chain details were entered. The PDB ID used was 6A93, the mutation details provided were I197V, and Chain A was identified to run the prediction. The stability of the protein can be quantitatively measured in terms of Gibbs free energy. The change of protein stability generated by nsSNPs to the wild type protein was calculated in terms of ΔΔG (ΔΔG = ΔGnsSNP – ΔGwt). The ΔΔG value was calculated for selected nsSNPs.

### Cell culture and maintenance

#### Cell lines and culture conditions

The MDA-MB-468 human breast cancer cell line, derived from a triple-negative breast cancer (TNBC) case in an AA woman, was obtained from the American Type Culture Collection (ATCC, Manassas, VA, USA). The non-tumorigenic MCF-10A human mammary epithelial cell line was kindly provided by Dr. Yasmine Kanaan (Howard University, Washington, DC, USA). Both cell lines were authenticated by short tandem repeat (STR) profiling and tested for mycoplasma contamination prior to use.MDA-MB-468 cells were cultured in RPMI-1640 medium supplemented with 10% fetal bovine serum (FBS) and 1% penicillin-streptomycin. MCF-10A cells were maintained in MEBM^™^ Basal Medium supplemented with bovine pituitary extract (BPE), human epidermal growth factor (hEGF), insulin, and 100 ng/mL cholera toxin, according to the manufacturer’s instructions. Cells were incubated at 37°C in a humidified atmosphere containing 5% CO_2_ and passaged at 85–90% confluency using phenol red-free TrypLE^™^ Express. Cell viability and density were assessed using trypan blue exclusion and a hemocytometer.

#### Cell viability and proliferation assays

MDA-MB-468 cells were seeded at 8,000 cells/cm^2^ in 96-well plates and incubated overnight. After serum starvation (0.5% FBS) for 24 h, cells were treated with (±)-DOI hydrochloride (Sigma-Aldrich) at concentrations of 1.25, 2.5, 5, 10, and 20 μM for 48 h. Untreated cells served as negative controls. Cell viability was assessed using the CellTiter 96^®^ AQueous One Solution Cell Proliferation Assay (Promega), with absorbance measured at 490 nm using a SpectraMax Plus microplate reader (Molecular Devices). Dose-response data were analyzed using a four-parameter logistic regression model via the AAT Bioquest EC_50_ calculator (https://www.aatbio.com/tools/ec50-calculator). EC_50_ values were derived from normalized triplicate data and validated by visual inspection of the fitted curve.

#### RNA isolation and miRNA expression analysis

Total RNA, including miRNA, was extracted from cultured cells using the AllPrep DNA/RNA Mini Kit (Qiagen), with a modified protocol to enrich for small RNAs. Specifically, 1.5 volumes of 100% ethanol were added to the flow-through in step 6. RNA concentration and purity were assessed using the NanoDrop^™^ One/OneC UV-Vis Spectrophotometer (Thermo Fisher Scientific). Samples were further purified using the Monarch Total RNA Miniprep Kit (NEB, #T2010) and stored at −80°C until use in NanoString nCounter assays. All experiments were conducted in biological triplicate with technical replication to ensure reproducibility. Multiplex miRNA profiling was conducted using the nCounter^®^ Human v3 miRNA Expression Assay (NanoString Technologies, Seattle, WA, USA), which detects 827 endogenous human miRNAs. Prior to hybridization, RNA samples were normalized to a concentration of 33 ng/μL. Following annealing and ligation, 100 ng of RNA was hybridized according to the manufacturer’s protocol. Hybridized samples were processed using the nCounter^®^ SPRINT Profiler (NanoString Technologies) to quantify miRNA expression levels.

### Statistical analysis

A comprehensive statistical and bioinformatic pipeline was employed to ensure robust analysis. Case-control associations between SNPs and breast cancer risk were evaluated using SNPStats (version 25H2) (https://www.snpstats.net/start.htm), applying logistic regression to calculate odds ratios (ORs) and 95% confidence intervals (CIs). Haplotype frequencies, Hardy-Weinberg equilibrium (HWE), and linkage disequilibrium (LD) were also assessed. Statistical significance was defined as p < 0.05, with Bonferroni correction applied for multiple comparisons to yield corrected p-values (Pc). Differentially expressed miRNAs were analyzed using nSolver^™^ Analysis Software (NanoString Technologies, version 4.0), which included quality control and normalization. Expression data from TNBC-treated, untreated, and control cells were averaged from triplicate experiments. miRNAs with p < 0.05 were considered significant. Overlapping miRNAs across experimental groups were visualized using Venn diagrams. To explore biological relevance, Ingenuity Pathway Analysis (IPA, Qiagen) was used to identify enriched pathways and molecular networks regulated by the differentially expressed miRNAs. Target validation and annotation were cross-referenced using miRBase (http://www.mirbase.org/), ensuring inclusion of only validated or high-confidence predicted interactions. This integrated approach ensured analytical rigor, reproducibility, and biological interpretability. The sample size provided adequate statistical power for detecting moderate-to-large effect sizes; however, analyses involving rare haplotypes may be underpowered.

## Results

### *In silico* analysis and SNP selection

The study identified a total of 2,291 SNPs within the HTR2A gene, sourced from the NCBI 1000 Genomes Browser Phase 3 dataset. From this pool, four SNPs (rs6304, rs6311, rs1745837, and rs7322347) were specifically chosen for further investigation. Among these, rs6304, rs1745837, and rs7322347 were prioritized due to their notable statistical significance (p < 0.00001) and variance in minor allele frequency (MAF), as outlined in Table 4. Noteworthy is the selection of rs6311, which, despite not exhibiting statistical significance (p = 0.335), was included based on its association with stress-related disorders, as documented in prior research. Of particular interest are the MAF disparities observed across populations. For instance, rs1745837 demonstrated the highest MAF within the AFR population, at 99.17%, while rs6304 exhibited the lowest MAF within the same population, standing at 7.56%. Intriguingly, the MAF for rs6304 among individuals of EUR descent was recorded as 0% according to data from the NCBI 1000 Genomes Browser Phase 3. This selection process underscores the meticulous attention paid to both statistical significance and population-specific allele frequencies, enhancing the precision and relevance of the subsequent analyses.

In our investigation of SNP functionality post-selection, we unveiled intriguing insights into their potential impact on the HTR2A and associated physiological processes. Within exon 3, SNP rs6304 presented a compelling case for functional significance. This SNP undergoes a consequential alteration from the major allele, T, to the minor allele, C, resulting in a noteworthy shift in the amino acid sequence. Specifically, this substitution replaces the major allele’s isoleucine (Ile) with valine (Val), introducing another hydrophobic branched-chain amino acid. Meanwhile, our attention turned to SNP rs6311, positioned intronically near the promoter region of HTR2A (Table 5). Our functional predictions hinted at its potential influence on gene expression, with a possible association with a transcription factor-binding site (TFBS) of HTR2A. This conjecture gains credibility from the regulatory score of 0.4496 attributed to rs6311, along with a calculated CADD score of 10.58. These findings deepen our understanding of how rs6311 may modulate HTR2A expression, potentially impacting various physiological pathways. Notably, among the intronic SNPs, rs1745837 stood out with a regulatory score of 0. Despite its intronic location, this SNP garnered attention in genetic association studies, notably linked to obesity [36]. Conversely, rs7322347, also intronic, displayed functional significance by disrupting a potential miRNA binding site, as elucidated by [37]. The reported regulatory score for rs7322347 was 0.054, further highlighting its possible impact on post-transcriptional gene regulation. Additionally, the assessment of conservation scores provided valuable insights into the evolutionary importance of these SNPs. Remarkably, rs6304 exhibited the highest conservation score among the selected SNPs at 0.997. This observation underscores its occurrence within a highly conserved and functionally critical genomic region, hinting at its potentially pivotal role in various biological processes. Overall, these findings contribute significantly to our understanding of how genetic variation within the HTR2A gene may influence gene expression and phenotype, shedding light on potential mechanisms underlying physiological and pathological processes.

### SNPs and haplotype association with BCa

The association between four HTR2A SNPs (rs6304, rs6311, rs1745837, and rs7322347) and the risk of BCa in AA women was assessed through a case-control study. The genotype and allele distributions for the selected SNPs were tested for Hardy-Weinberg Equilibrium in both case and control groups. Odds ratios (ORs) greater than 1 indicate increased risk, whereas ORs less than 1 indicate a protective effect. Some large odds ratios observed in haplotype analyses (e.g., OR > 100) likely reflect low-frequency combinations and small subgroup sizes and should therefore be interpreted with caution. A summary of the genotype and allele frequencies between BCa cases and controls is presented in Table 6. For rs6304, associations varied depending on the genetic model. The heterozygous genotype was associated with increased risk in the overdominant model, whereas the dominant model suggested a protective effect. The codominant model showed no statistically significant association. These differences likely reflect model-specific effects and should be interpreted cautiously (Table 6).

For rs1745837, a strong association with breast cancer risk was observed across multiple genetic models. In particular, the heterozygous genotype showed a significantly increased risk in the overdominant, dominant, and codominant models. These findings consistently indicate that rs1745837 is associated with increased susceptibility to BCa in this population (Table 6). For rs7322347, no significant association with BCa risk was observed across the various genetic models

### Assessment of rs6304’s impact on HTR2A stability

**Figure 2.**
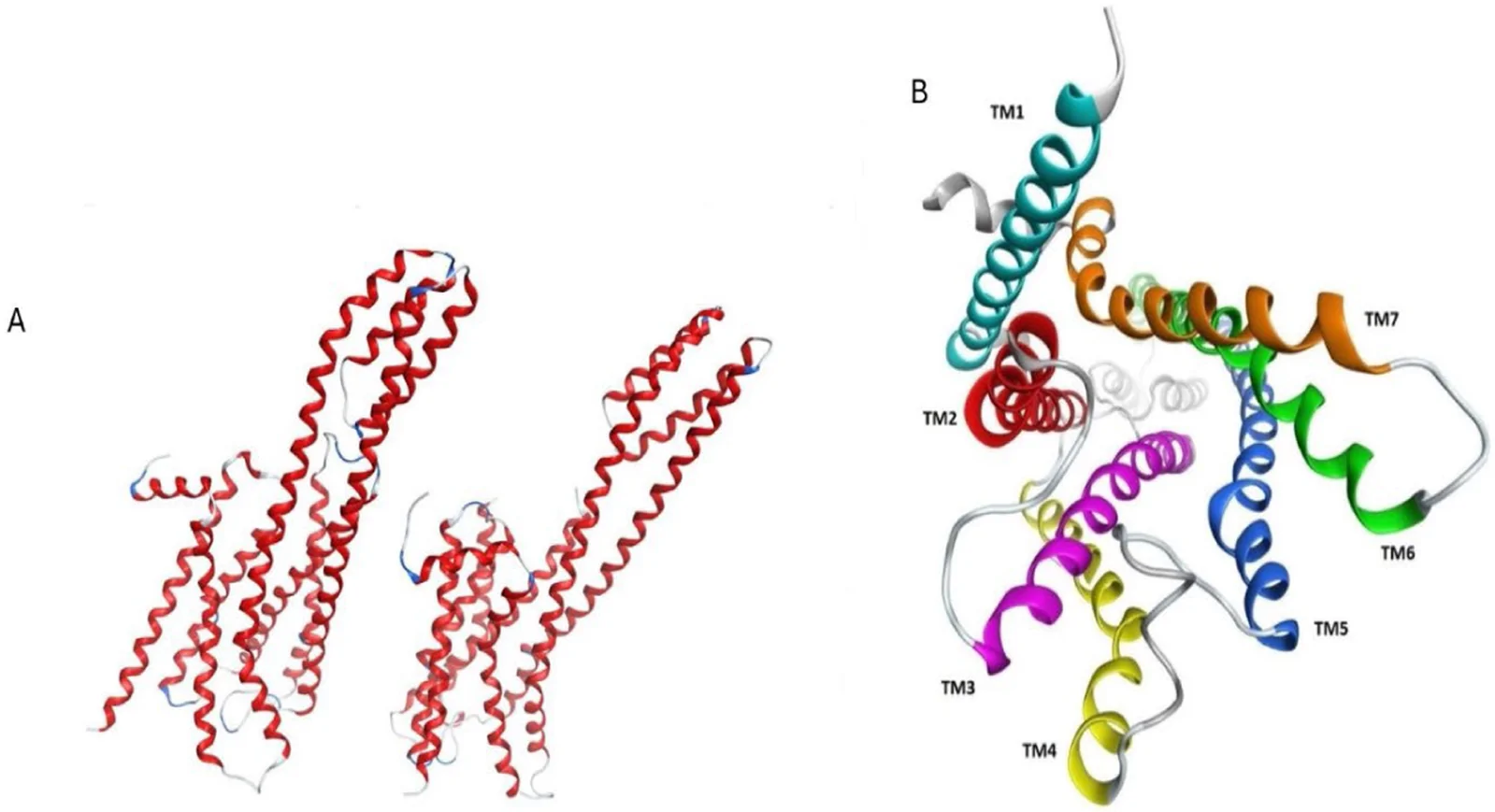
Molecular Modeling Analysis of rs6304’s Impact on HTR2A Stability. **A**) Dimeric structure of HTR2A without zotepine and zinc ions, serving as the baseline for further structural analyses. **B**) X-ray crystal structure of HTR2A, illustrating its GPCR architecture with seven transmembrane helices (TM1–7) and intracellular and extracellular loops.

**Figure 3.**
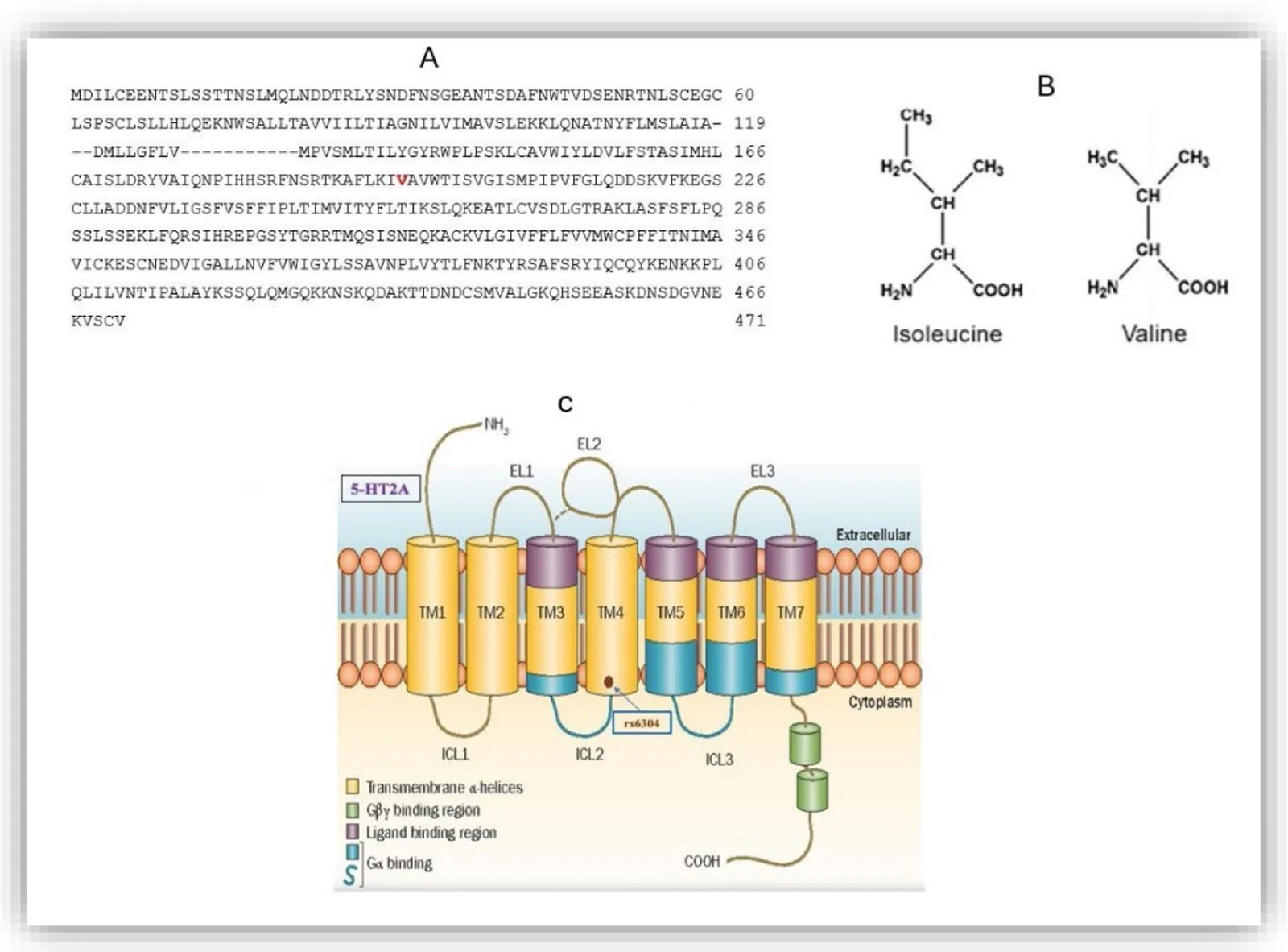
Molecular Insights into SNP rs6304. **A**) Molecular depiction highlighting the neighboring amino acids, isoleucine, and alanine, flanking residue 197, influenced by SNP rs6304. **B**) Enhanced view focusing on residue 197 within exon 3, showcasing the non-synonymous change from isoleucine to valine induced by SNP rs6304. **C**) Schematic representation of HTR2A indicating the position of rs6304. Shaded regions within the intracellular environment denote areas hypothesized to interact with G-proteins [38].

**Figure 4.**
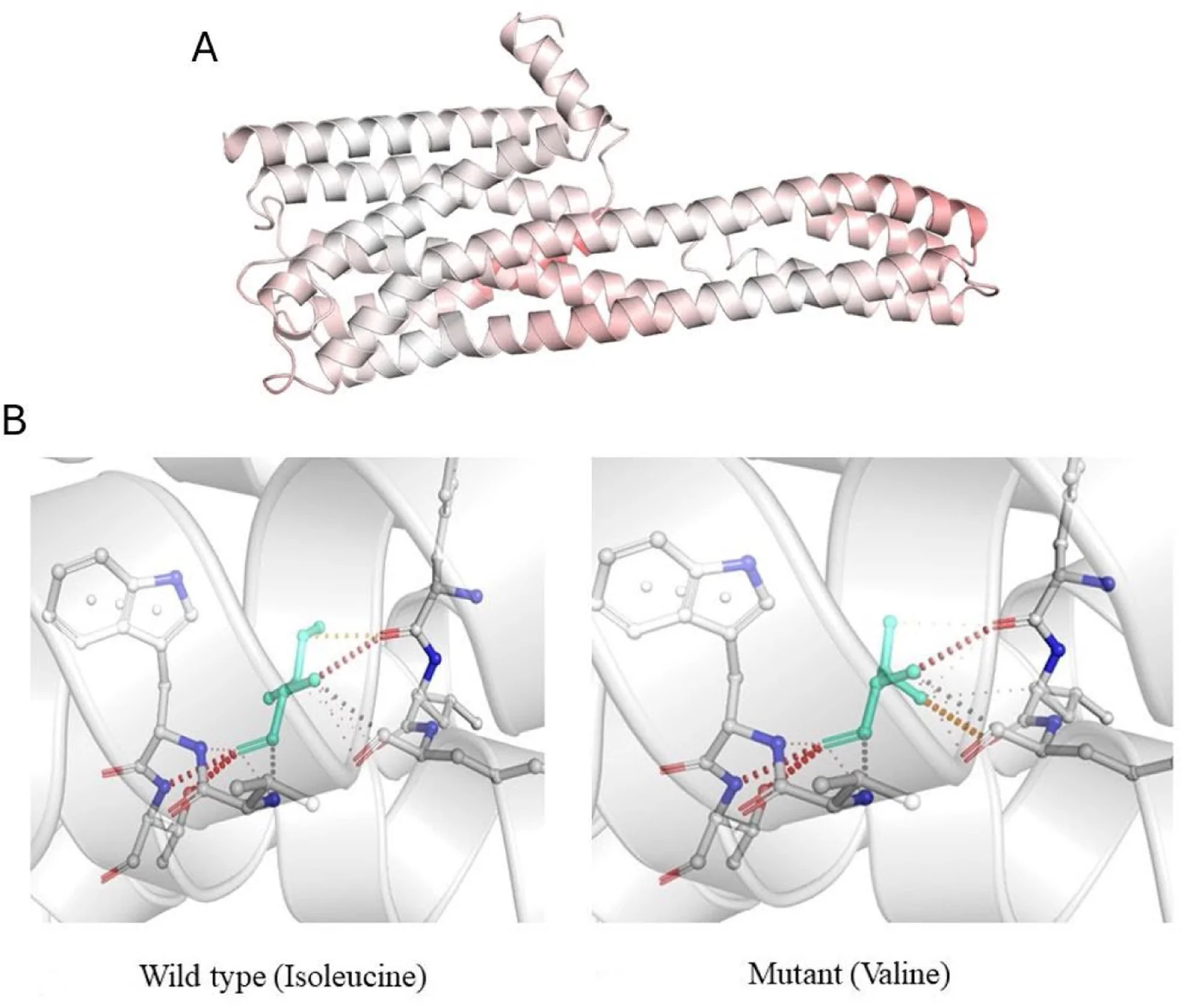
Vibrational Entropy Energy and Interaction Changes Induced by rs6304. A) Representation of vibrational entropy energy within HTR2A affected by rs6304. Regions colored in red indicate gained flexibility due to the mutation. B) Visualization of interatomic interaction changes with neighboring residues in HTR2A caused by rs6304 mutation. Wild-type residues are depicted in light green, while mutant residues, displaying alterations due to the missense variation, are shown in the same color. Both types of residues are represented as sticks alongside surrounding residues engaged in diverse interactions. Hydrogen bonds are highlighted in red, while weaker hydrogen bonds are depicted in orange, providing insights into the intricate molecular contacts within the protein structure.

Molecular modeling was employed to investigate the impact of rs6304, a non-synonymous single-nucleotide polymorphism, on the stability of HTR2A. Data sourced from the National Center for Biotechnology Information (NCBI) and Protein Data Bank (PDB) facilitated this analysis, focusing on the variant 1 isoform of HTR2A, consisting of 471 amino acids. Utilizing the available crystal structure of human HTR2A in complex with zotepine, an antipsychotic drug (PDB ID: 6A94), obtained from the PDB, molecular modeling was initiated using Molecular Operating Environment (MOE). This crystal structure revealed a dimeric arrangement of HTR2A, with one receptor unit containing a bound zinc ion within its asymmetric unit. Figure 2A depicts the dimeric structure of HTR2A devoid of zotepine and zinc ions, serving as the baseline for further structural analyses. The X-ray crystal structure of HTR2A delineates its characteristic architecture as a G protein-coupled receptor (GPCR), comprising seven transmembrane helices (TM1–7) with an extracellular amino terminus and an intracellular carboxyl terminus, as depicted in Figure 2B. These helices are interconnected by intracellular and extracellular loops, embodying the structural basis for ligand recognition and signal transduction. The structural organization of HTR2A, including its seven transmembrane domains and connecting loops, is summarized in Table 8. Central to HTR2A’s functionality are three critical motifs implicated in ligand-induced conformational changes and downstream signaling pathways, as detailed in Table 9. Notably, the DRY motif within intracellular loop 2 (IL2), the NPxxY motif in transmembrane helix 7, and the PDZ-binding motif in the cytoplasmic C-terminus orchestrate receptor activation and cellular responses. Our investigation focused on SNP rs6304, located on exon 3, which induces a non-synonymous alteration from isoleucine to valine at residue 197 (Figure 3B), flanked by isoleucine and alanine (Figures 3A and 3C). Both amino acids possess branched side chains and are non-polar, uncharged, and hydrophobic, with valine’s side chain comprising an isopropyl group. Thermodynamic analyses consistently demonstrated that the rs6304 variant exerts a destabilizing effect on the HTR2A protein. Multiple prediction tools yielded negative ΔΔG values, indicating reduced structural stability (Table 10). Additionally, vibrational entropy analysis revealed increased molecular flexibility, suggesting that the mutation may alter receptor dynamics and potentially impact signaling. Furthermore, we investigated the impact of rs6304 on protein dynamics and thermostability through vibrational entropy analysis, revealing a ΔΔSVib ENCoM of 0.125 kcal/mol K, suggesting increased molecular flexibility (Figure 4A). Examination of interatomic interactions highlighted alterations in hydrogen bonding upon substitution of isoleucine with valine, as depicted in Figure 4B. These findings illuminate the molecular mechanisms underlying the destabilizing effect of rs6304 on HTR2A stability, providing valuable insights into its functional implications and potential therapeutic targets.

### Cell viability and proliferation assays

**Figure 5.**
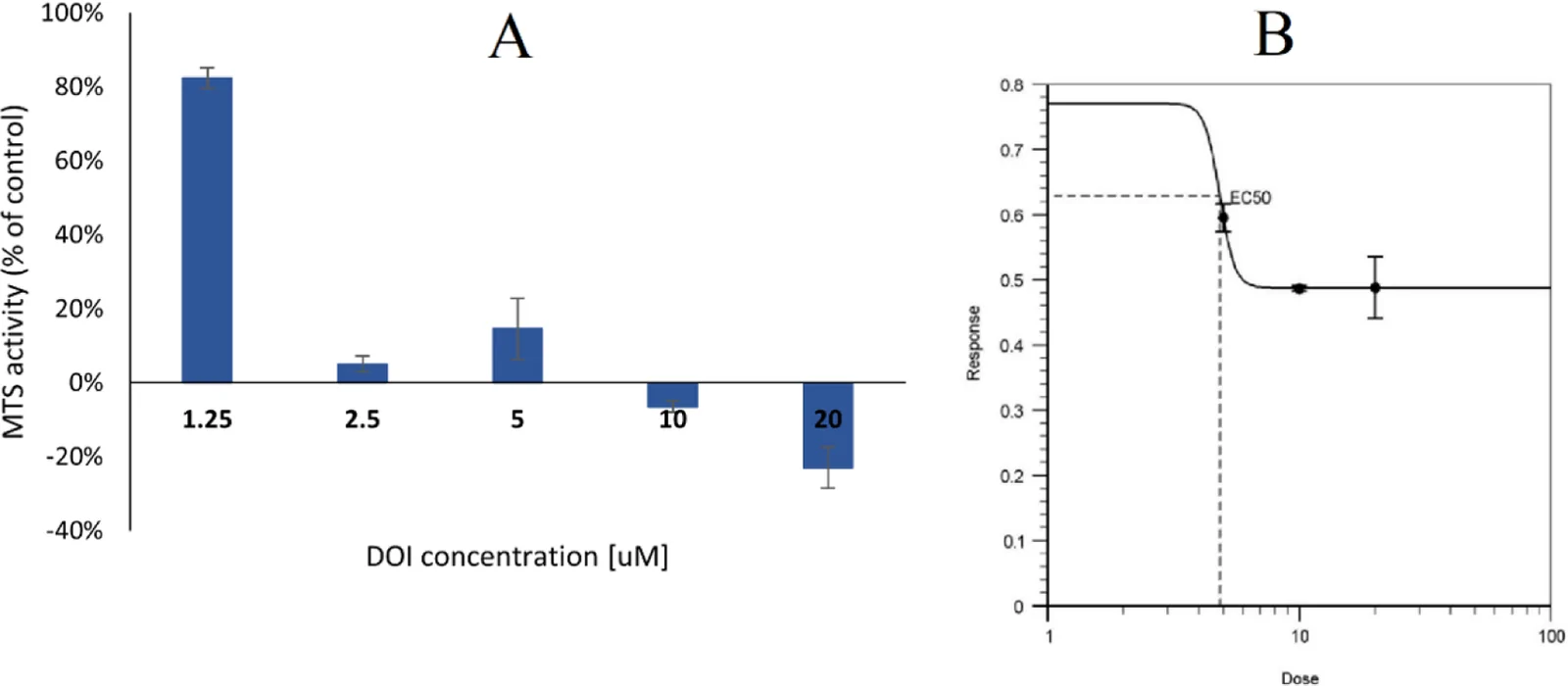
Effect of 5-HT2A receptor activation on TNBC cell proliferation. (**A**) Dose-dependent response of MDA-MB-468 cells to DOI treatment, expressed as percentage of control. (**B**) Dose–response curve used to determine EC50. Error bars represent the standard error of the mean from triplicate experiments.

The MTS Cell Proliferation Assay was performed to determine the effect of HTR2A biological activity on MDA-MB-468 cell lines. Cells were plated and incubated for 48 hours with varying concentrations of DOI (Millipore Sigma), an agonist of HTR2A. The concentrations of DOI used were 1.25, 2.5, 5, 10, 20 μM. Cell proliferation was determined spectrophotometrically, and the average cell proliferation effect was calculated. Considerable proliferation was observed at 1.25 μM in MDA-MB 468 at 82% higher compared to the control, untreated cells in 0.5% serum conditions (Figure 5A). The results of this experiment demonstrated that HTR2A has a proliferative effect on cell line MDA-MB-468. To determine EC50 of MDA-MB 468, the maximal activity in MDA-MB 468 cells occurred at 1.25μM. The EC50 value was 5 μM (Figure 5B).

### Cell lines’ genotyping

Cell lines MCF-10A and MDA-MB-468 were genotyped for specific HTR2A SNPs to identify potential genetic variations linked to breast cancer risk, as shown in Table 11. Both cell lines were heterozygous (C/T) for rs6304. For rs6311, MCF-10A was homozygous for the minor allele T (T/T), whereas MDA-MB-468 was homozygous for the major allele (C/C). Neither cell line carried the minor allele T of rs1745837, with both displaying the homozygous C/C genotype. Notably, MDA-MB-468 was homozygous for the minor allele T (T/T) of rs7322347, a SNP associated with miRNA binding, while MCF-10A was heterozygous (A/T). Importantly, MDA-MB-468 cells were found to carry the TCCT haplotype, previously identified in this study as being associated with an increased risk of breast cancer. Importantly, MDA-MB-468 cells carry the TCCT haplotype associated with increased breast cancer risk and exhibit homozygosity for the rs7322347 variant, which has been linked to altered miRNA binding (Table 11). These features support the use of this cell line as a relevant model for investigating HTR2A-mediated miRNA regulation in TNBC.

### Differential expression profiles of miRNAs in TNBC

**Figure 6.**
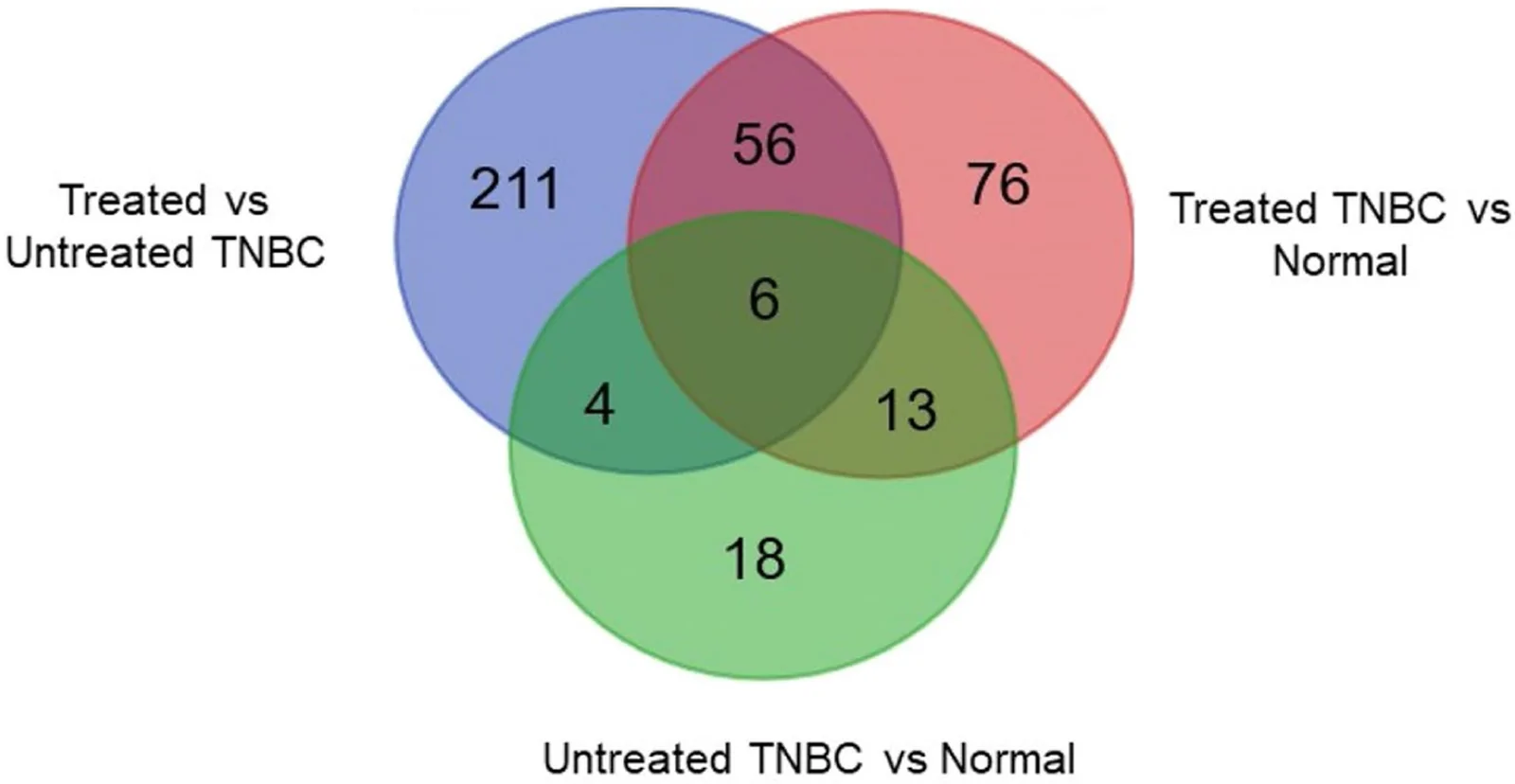
Identification of differentially expressed miRNAs in TNBC. Venn diagram illustrating overlaps of significantly dysregulated miRNAs across three comparisons: treated TNBC vs untreated TNBC, treated TNBC vs normal cells, and untreated TNBC vs normal cells. Six miRNAs were consistently dysregulated across all conditions.

**Figure 7.**
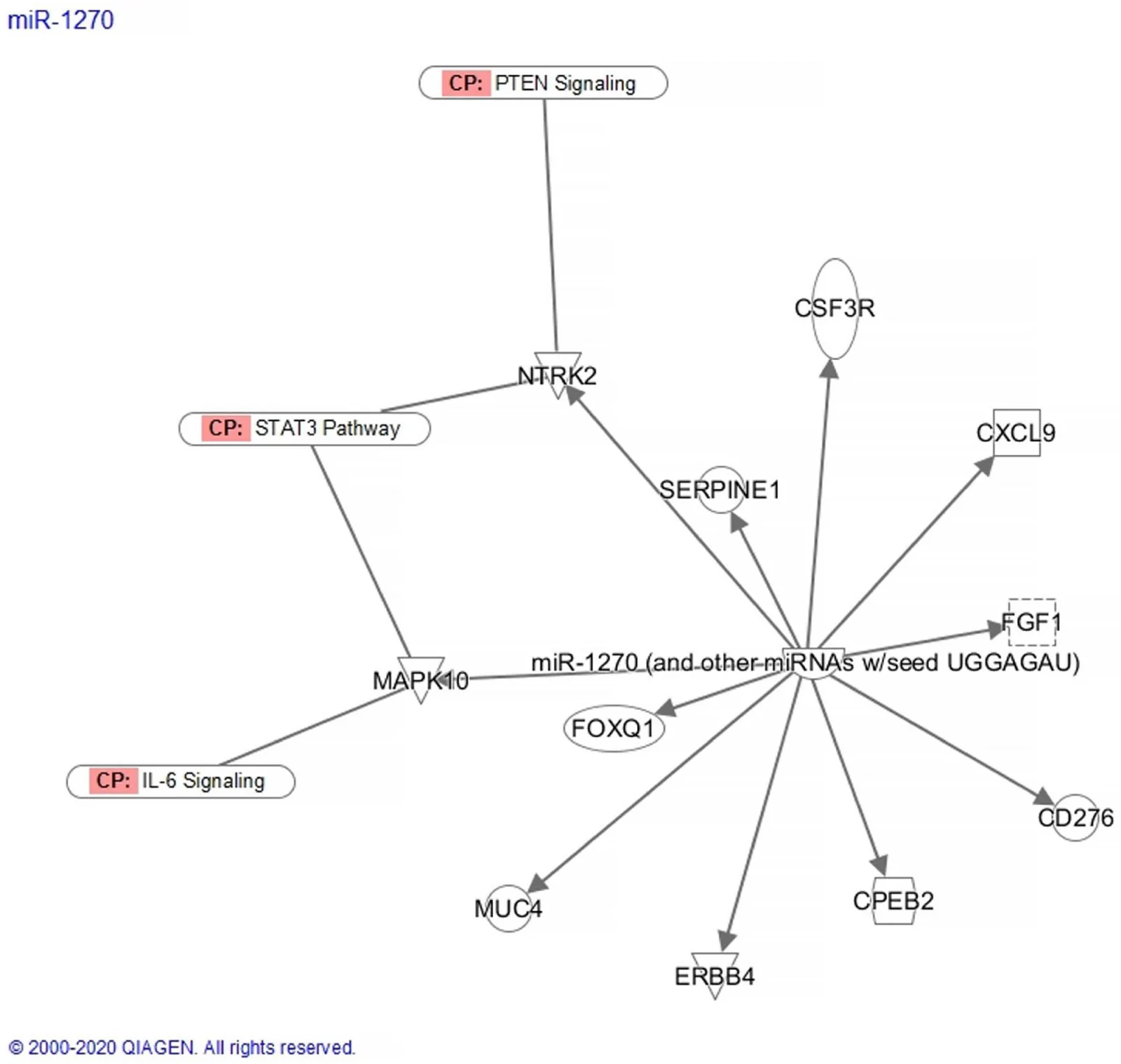
Regulatory network of miR-1270 in TNBC. Ingenuity Pathway Analysis of miR-1270 and related microRNAs sharing the seed sequence “UGGAGAU.” Circular nodes represent target genes, while rectangular nodes denote enriched canonical pathways. miR-1270 targets genes involved in key oncogenic pathways, including IL-6 signaling, STAT3 signaling, and PTEN-related pathways, highlighting its potential role in promoting tumor progression.

**Figure 8.**
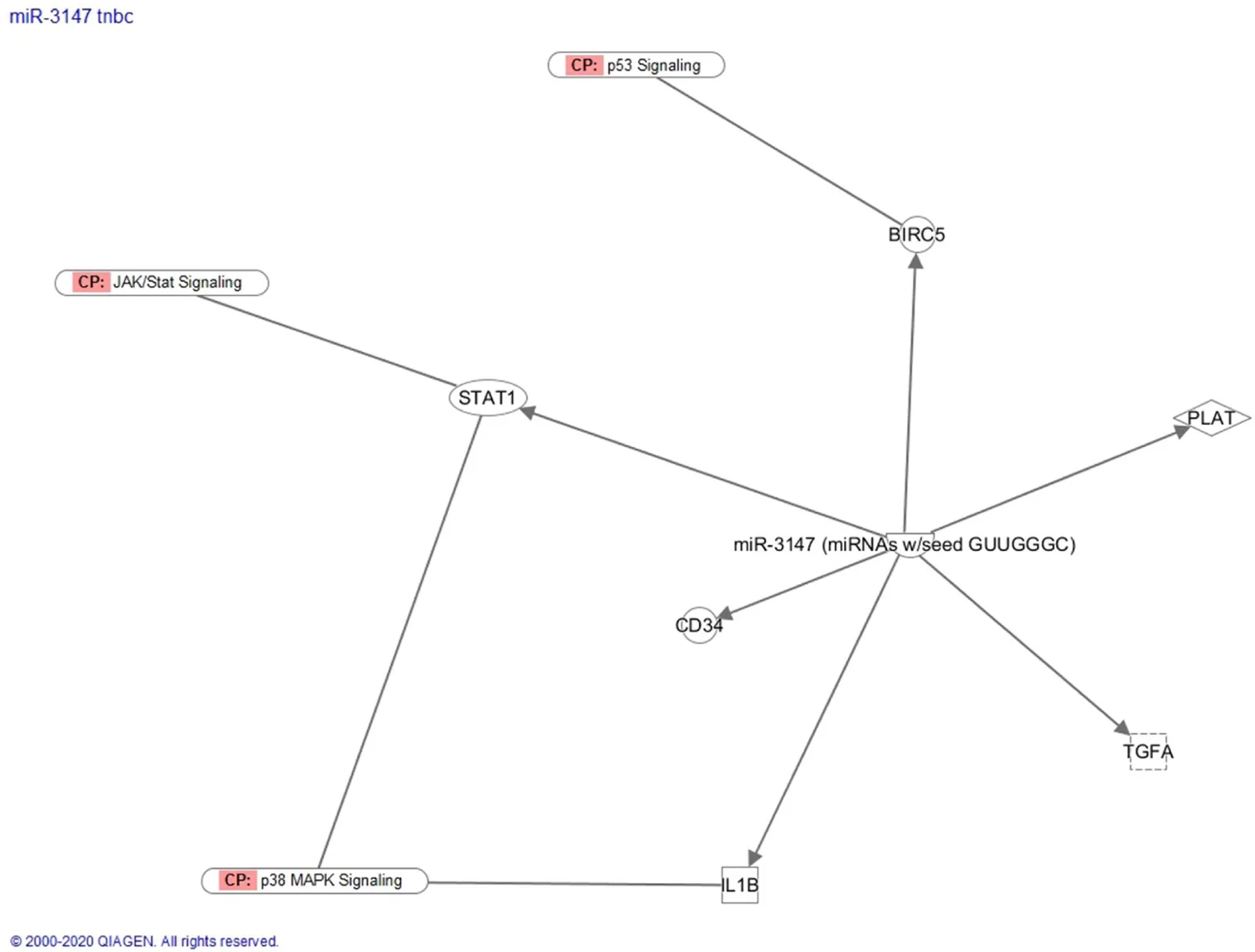
Regulatory network of miR-3147 in TNBC. Network generated using IPA demonstrating interactions between miR-3147 and its predicted target genes. Key pathways influenced include JAK/STAT, p53 signaling, and p38 MAPK signaling. These interactions suggest a role for miR-3147 in regulating cell proliferation, apoptosis, and metastatic processes in TNBC.

**Figure 9.**
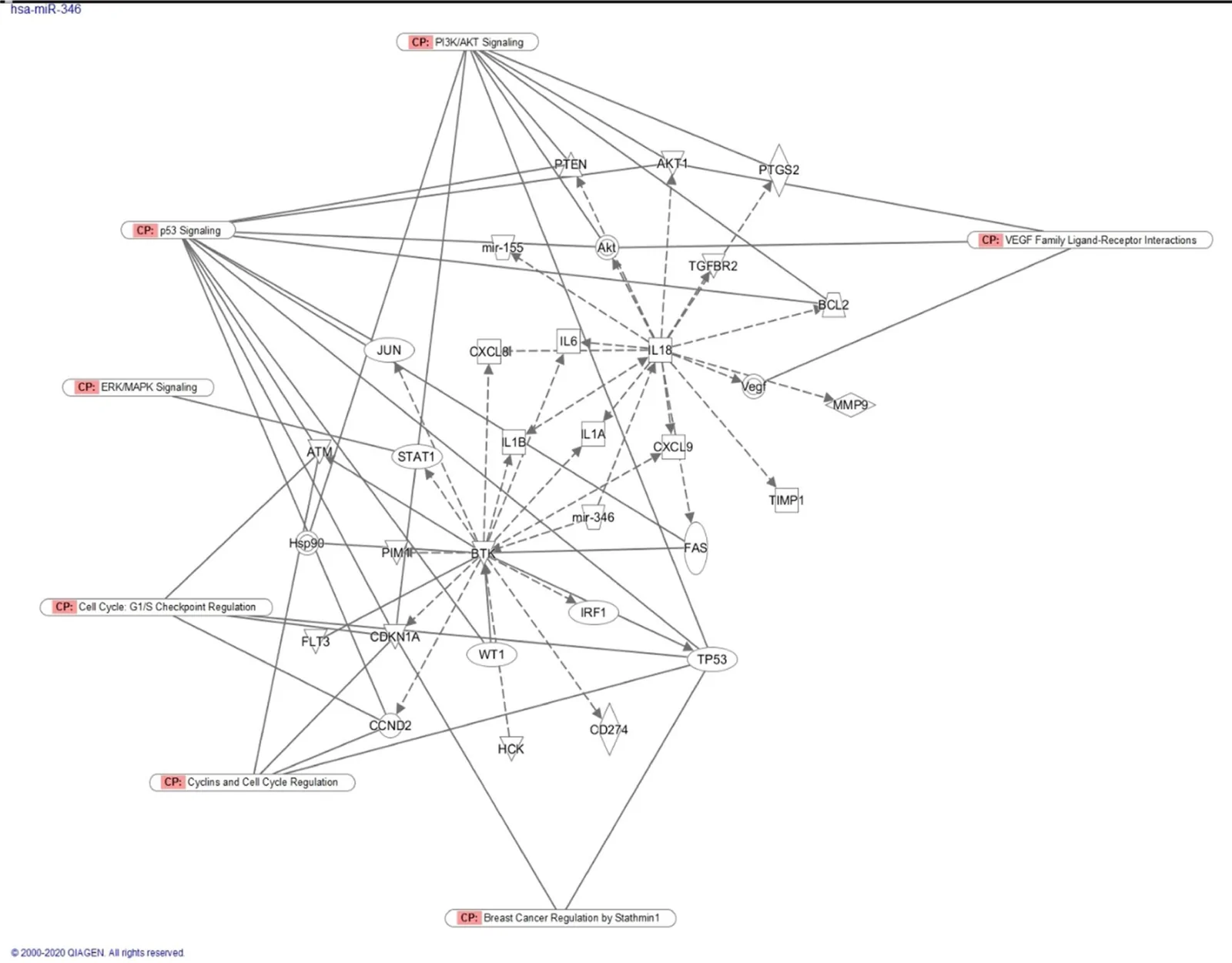
Regulatory network of miR-346 in TNBC. IPA-derived network illustrating the interactions of miR-346 with target genes involved in cancer-relevant pathways. Key pathways include PI3K/AKT signaling, ERK/MAPK signaling, VEGF signaling, and p53-mediated processes. The network highlights the potential role of miR-346 in tumor progression, angiogenesis, and survival signaling.

**Figure 10.**
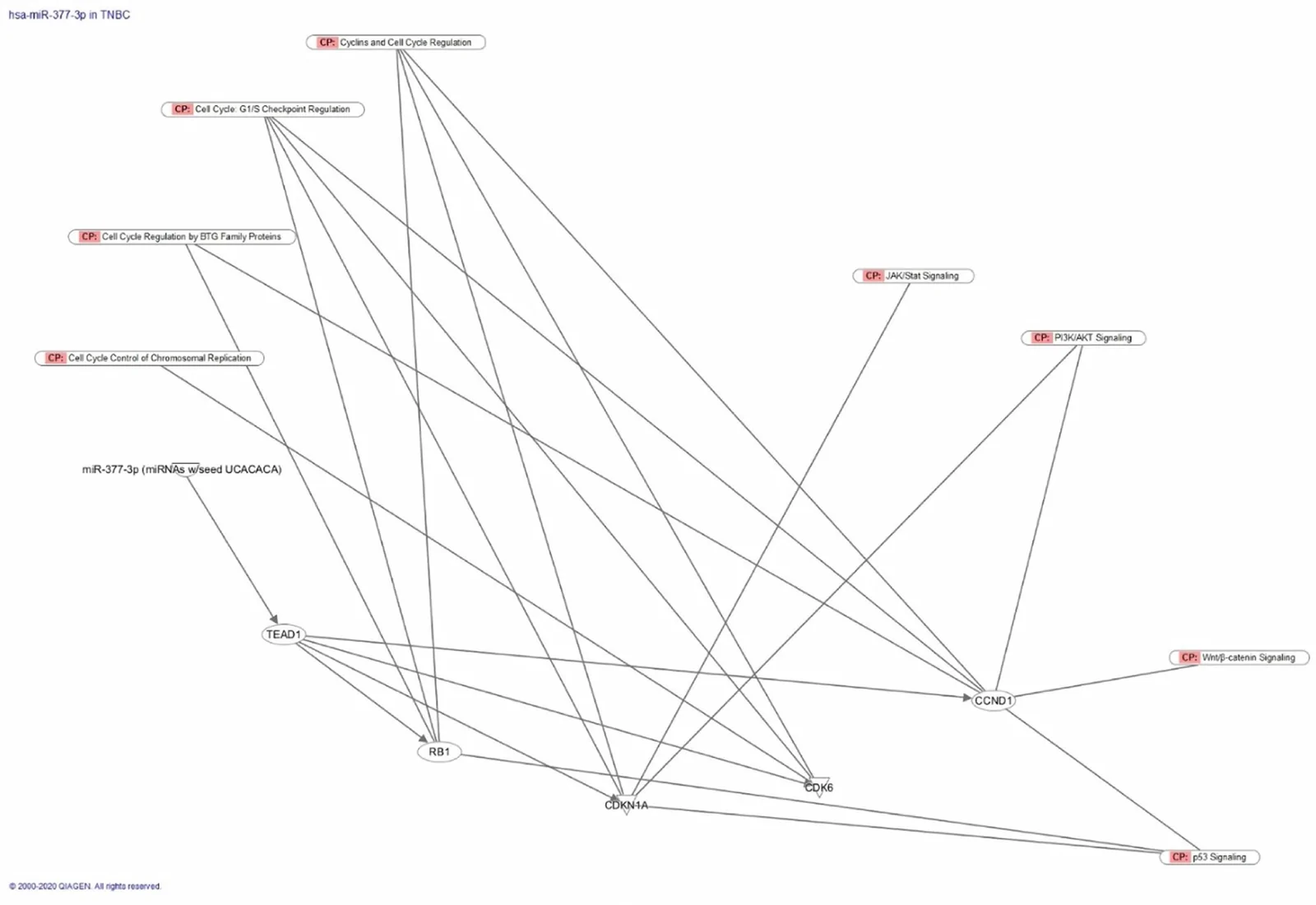
Regulatory network of miR-377–3p in TNBC. Network analysis showing the regulatory impact of miR-377–3p on genes associated with cell cycle progression and oncogenic signaling. Target genes include CDK6, CCND1, and RB1, influencing pathways such as PI3K/AKT, JAK/STAT, and p53 signaling. These findings support a role for miR-377–3p in promoting tumor growth and cell cycle dysregulation.

**Figure 11.**
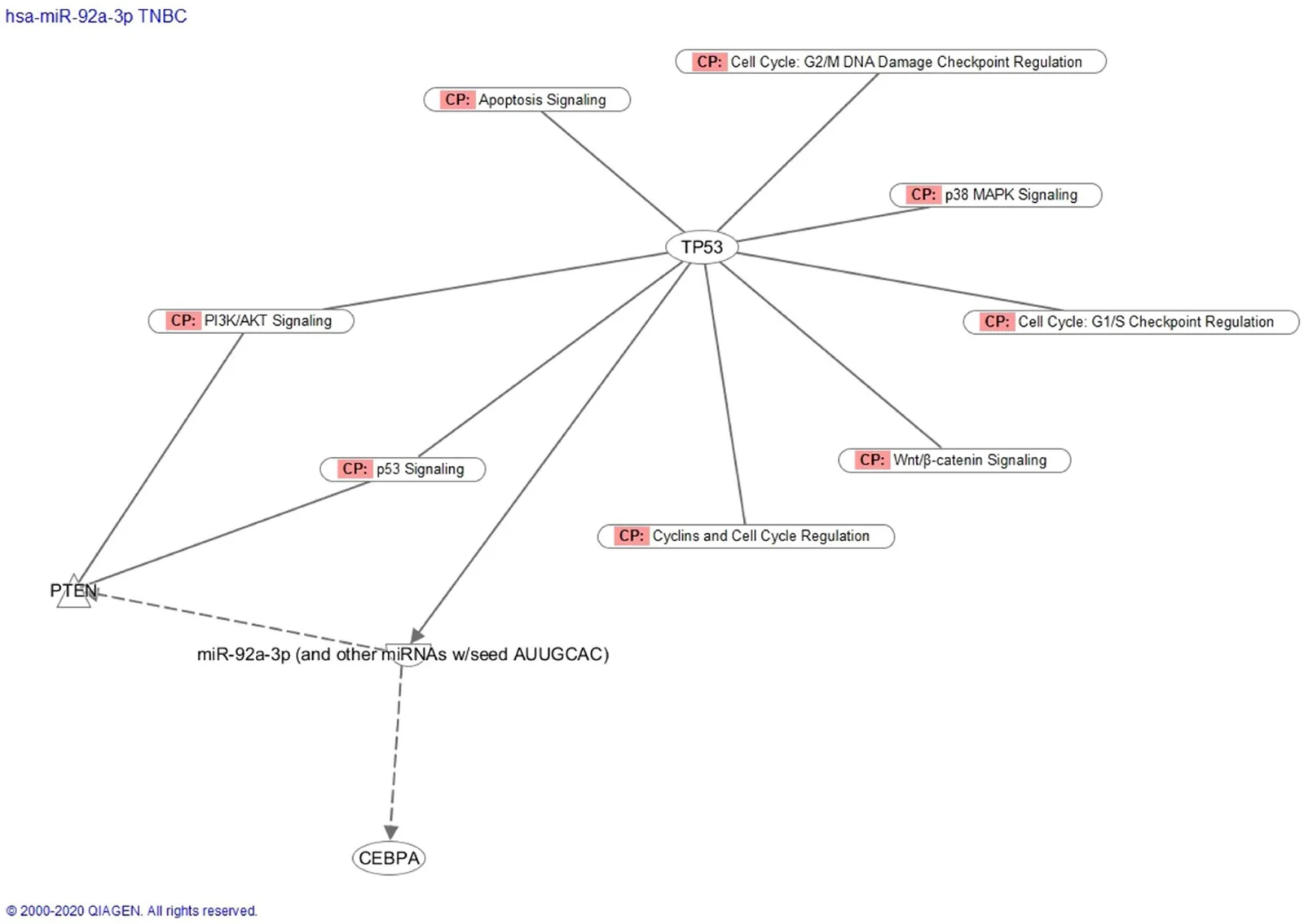
Regulatory network of miR-92a-3p in TNBC. IPA network illustrates interactions between miR-92a-3p and key tumor suppressor genes, including PTEN and TP53. The network highlights involvement in PI3K/AKT signaling, apoptosis, and cell cycle regulation, consistent with the oncogenic role of miR-92a-3p in promoting tumor progression and therapeutic resistance.

**Figure 12.**
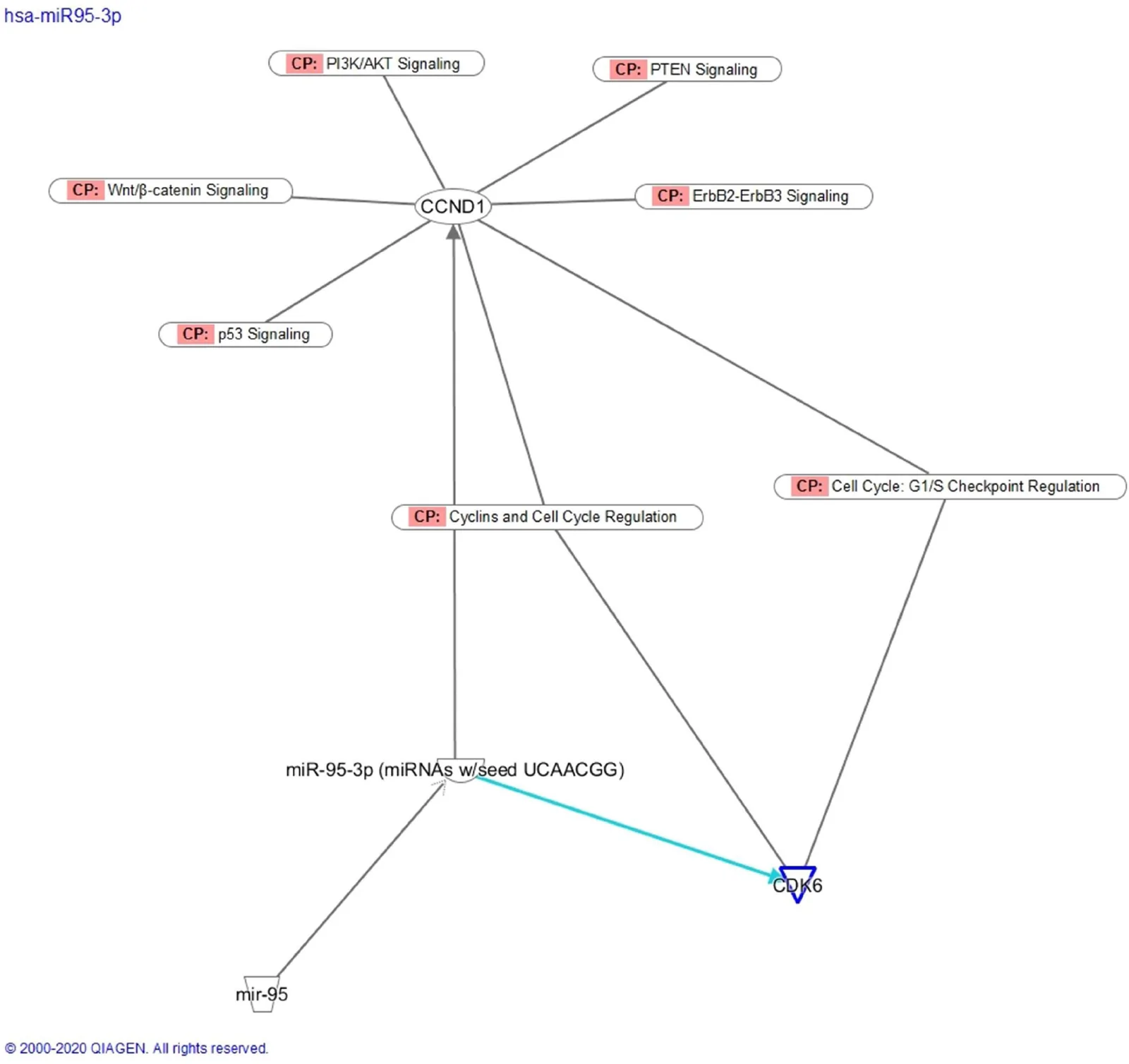
Regulatory network of miR-95–3p in TNBC. Network visualization demonstrating the regulatory interactions of miR-95–3p with genes involved in cell cycle control and oncogenic pathways. Key targets include CDK6 and CCND1, with associated pathways including PI3K/AKT signaling, Wnt/β-catenin signaling, and p53 signaling. These findings suggest that miR-95–3p contributes to tumorigenesis through modulation of cell proliferation and survival pathways.

To investigate miRNA expression profiles in TNBC, we utilized the human breast cancer cell line MDA-MB-468 and the non-tumorigenic mammary epithelial cell line MCF-10A. MDA-MB-468, characterized as TNBC due to its lack of ER, PR, and HER2 expression, was selected for its TCCT haplotype and homozygosity for the minor allele T of rs7322347, which is significantly associated with miRNA binding. Our breast cancer association study confirmed that the TCCT haplotype is linked to increased susceptibility to breast cancer. MCF-10A cells were used as a control representing normal human mammary epithelial cells. miRNA expression levels were quantified in DOI-treated MDA-MB-468, untreated MDA-MB-468, and MCF-10A cells using the nanoString nCounter platform, which targets 827 human miRNAs. DOI (2,5-Dimethoxy-4-iodoamphetamine), an agonist of the HTR2A, was used for its known pharmacological effects. MDA-MB-468 cells were cultured in media with limited fetal bovine serum (FBS), both with and without DOI treatment. The untreated MDA-MB-468 and MCF-10A cells were cultured in similar conditions for comparison. Differential expression analysis identified six miRNAs that were consistently dysregulated across all comparisons, and these are summarized in Table 12. Statistical analysis was performed using nSolver software, employing a t-test to determine significance. Differentially expressed miRNAs (p < 0.05) were identified and visualized using a Venn diagram (Figure 6), to compare treated vs untreated TNBC, treated TNBC vs normal cells, and untreated TNBC vs normal cells. Six miRNAs (miR-1270, miR-3147, miR-346, miR-377–3p, miR-92a-3p, and miR-95–3p) were consistently upregulated across all comparisons and were classified as oncogenic based on their known targets and biological functions (Table 12). Bioinformatic analysis and Ingenuity Pathway Analysis (IPA) were conducted to identify key targets and pathways regulated by these miRNAs. The results, summarized in Table 12, demonstrate that these miRNAs primarily target genes involved in cell cycle regulation, apoptosis, and inflammatory signaling. IPA analysis revealed that miR-1270 targets include Mitogen-activated Protein Kinase 10 (MAPK10) and Neurotrophic Receptor Tyrosine Kinase 2 (NRTK2), impacting Interleukin 6 (IL-6) Signaling. MiR-95–3p was identified as targeting Signal Transducer and Activator of Transcription 1 (STAT1) and the p38 Mitogen-Activated Protein Kinase (MAPK) Signaling pathways. Additionally, miR-346 was suggested to influence Interleukin 18 (IL-18) and Bruton’s tyrosine kinase (BTK) indirectly. Both miR-377–3p and miR-92a-3p targeted Cyclin Dependent Kinase 6 (CDK6), among other pathways, potentially promoting cancer progression. Furthermore, miR-92a-3p was found to downregulate Phosphatase and Tensin Homolog (PTEN). Canonical pathways for each oncogenic miRNA are depicted in Figures 7–12. Notably, several of the identified miRNAs (e.g., miR-92a-3p and miR-377–3p) target key regulators such as PTEN and CDK6, suggesting a mechanistic link between HTR2A genetic variation and oncogenic signaling pathways.

## Discussion

This study demonstrates that genetic variation in HTR2A is associated with breast cancer susceptibility in African American women and is linked to altered miRNA expression in TNBC models. These findings collectively suggest that serotonergic signaling pathways may contribute to tumor progression through coordinated genetic and epigenetic mechanisms. Genetic variation in cancer susceptibility is a well-recognized phenomenon [39]. Recent advancements in genomics have identified SNPs as potential biomarkers for cancer diagnosis and treatment [40]. However, the association between SNPs in the HTR2A gene and BCa has been scarcely investigated. Although HTR2A genetic variation has been explored in other diseases [41–43], its role in BCa remains largely unexplored. The choice of the selected SNPs was based on several criteria, including minor allele frequency differences between AFR and EUR populations, predicted deleteriousness, and known regulatory effects. SNPs with significant frequency differences, such as rs1745837 and rs7322347, were prioritized, while rs6304 and rs6311 were selected due to their potential functional impact [44]. Additionally, regulatory potential scores from NEIHS tools and FuncPred predictions supported the functional relevance of rs6304, rs6311, and rs7322347. Notably, rs6311’s impact on transcription factor binding and rs7322347’s effect on miRNA binding highlight their potential roles in gene expression regulation [44,45]. Conservation scores further reinforced the functional importance of rs6304, indicating its presence in a conserved genomic region [44–46]. This, combined with the predicted destabilizing effects of the rs6304 missense mutation on the HTR2A protein, underscores its potential impact on protein function. The Gibbs energy difference calculation by Dynamut revealed a destabilizing effect for rs6304, which is mapped to the transmembrane domain TM4 near the intracellular loop ICL2—crucial for G-protein coupling, aligning with findings in glioblastoma research, where cancer-related missense mutations often lead to destabilized protein structures [44–47]. Our findings align with the broader literature, suggesting that SNPs can significantly affect protein stability and function, contributing to disease pathology [47–49]. The consistent destabilizing effect of rs6304 across multiple computational models supports its potential functional relevance, particularly given its location near key regions involved in receptor signaling. This observed destabilization in HTR2A due to rs6304 highlights the need for further investigation into how serotonin-related genetic variations influence cancer progression.

Previous studies have shown that genetic variations in G protein-coupled receptors (GPCRs) can disrupt their function [50]. Although our study did not assess the binding affinity of the I197V mutation, it is an important consideration. This missense mutation occurs in TM4, near the intracellular loop ICL2, which is crucial for G-protein binding and downstream signaling [51]. Davies et al. (2006)[52] reported that I197V affects binding affinity, which might help explain our association data for rs6304 with BCa. Our study found significant associations between three SNPs (rs6304, rs6311, and rs1745837) and BCa. Myers et al. (2007) [53] demonstrated that rs6311 impacts HTR2A promoter activity, potentially linking the C/T genotype with increased BCa risk. Furthermore, rs1745837’s predicted effect on a miRNA binding site may influence gene expression and tumor growth by disrupting RNA silencing [54]. To our knowledge, this is the first study to investigate HTR2A genetic variation in BCa. Existing research has established serotonin’s role in cancer progression [55–57]. Our findings indicate that genetic variations in HTR2A, particularly rs6311 and rs1745837, are associated with BCa risk in AA. Notably, the BCa risk increased with the over-dominant inheritance model of rs6304. Most SNPs are considered biological markers rather than direct disease causes. Our study revealed haplotypes associated with increased BCa risk and identified linkage disequilibrium among the SNPs, particularly between rs6304, rs6311, and rs1745837. CADD scores indicated that rs6304 and rs6311 are likely deleterious: rs6304 with rs6311 and rs1745837, and rs6311 with rs1745837 and rs7322347. While our statistical analyses identified significant associations, we caution that large odds ratios (e.g., OR > 10) may reflect sampling variation, particularly for rare haplotypes. These results should therefore be interpreted with biological plausibility in mind, rather than statistical significance alone. The Oncomine database also supports HTR2A expression in BCa cells [58]. Based on our haplotype analysis, we examined miRNA profiles in the MDA-MB-468 TNBC cell line. The heterozygosity for rs6304 and the TCCT haplotype, associated with increased BCa risk, guided us to study HTR2A activation. We applied the Bonferroni correction to control Type I error, but we recognize this method is conservative and may increase false negatives. Future studies with larger cohorts may benefit from false discovery rate (FDR) approaches to balance sensitivity and specificity. We used DOI hydrochloride, an HTR2A agonist, to assess its mitogenic effects. Our results demonstrated increased cell proliferation in treated MDA-MB-468 cells, consistent with findings in MCF-7 cells [59]. Oufkir *et al*. [60,61] characterized the signaling pathways related to HTR2A activation in trophoblast choriocarcinoma cells, highlighting the role of JAK2, STAT3, and ERK1/2 in cell growth and survival. Given that HTR2A is well-characterized in other cancer types but not in TNBC, we chose to activate rather than inhibit HTR2A in our experiments.

Our study investigated the miRNA profile mediated by HTR2A, focusing on miRNAs involved in cell cycle progression and apoptosis. miRNAs can be classified as oncogenic or tumor-suppressing; oncogenic miRNAs inhibit tumor suppressor genes, while tumor-suppressing miRNAs target oncogenes. We identified six statistically significant differentially expressed miRNAs: has-miR-1270, hsa-miR-92a-3p, hsa-miR-377–3p, hsa-miR-95–3p, and hsa-miR-3147. Further analysis revealed that these miRNAs are oncogenic. Ingenuity Pathway Analysis (IPA) highlighted that hsa-miR-1270 targets MAPK10, a tumor suppressor involved in the STAT3 pathway and IL-6 signaling [62]. Upregulation of has-miR-1270 was observed in treated TNBC, aligning with its role as an oncogenic miRNA [63,64]. hsa-miR-3147 targets BIRC5 and STAT1, genes associated with cell proliferation and metastasis [65]. We observed increased levels of hsa-miR-3147 in treated TNBC compared to untreated cells. IPA’s analysis of hsa-miR-346 revealed indirect targeting of IL-18 and BTK, but not SRCIN1. These findings are supported by a study that showed overexpression of miR-346 promoted cell proliferation, colony formation, migration, and invasion, and reduced apoptosis [66]. Despite this, hsa-miR-346 was overexpressed in treated TNBC, indicating its oncogenic role; however, further validation is needed. hsa-miR-377 was predicted to target TEAD1, CDK6, CCND1, and RB1, all involved in cell cycle regulation [67]. Upregulation of hsa-miR-377 in TNBC suggests it may act as a biomarker due to its potential in tumorigenesis.hsa-miR-92a-3p, upregulated in treated TNBC, is linked to tamoxifen resistance and targets PTEN, a tumor suppressor that modulates the PI3K/Akt pathway [68,69]. Finally, hsa-miR-95–3p is known to repress CDKN1A and targets CDK6 and CCND1 [70]. Our results show upregulation of hsa-miR-95–3p, confirming its role as an oncogenic miRNA. The regulatory networks of hsa-miR-1270, hsa-miR-3147, hsa-miR-346, hsa-miR-377–3p, hsa-miR-92a-3p, and hsa-miR-95–3p demonstrate the intricate roles of microRNAs (miRNAs) in modulating key signaling pathways, particularly in the context of triple-negative breast cancer (TNBC). These networks collectively highlight common pathways, such as cell cycle regulation, PI3K/AKT signaling, p53 signaling, Wnt/β-catenin signaling, and apoptosis, which are hallmarks of cancer progression. A common theme across these miRNAs is their regulatory impact on oncogenic pathways and tumor suppressor genes, underscoring their dual roles as both oncogenes and tumor suppressors depending on the cellular context. Our findings are consistent with large-scale breast cancer miRNA profiling studies. For example, Khanabdali et al. (2025)[71] identified circulating miRNA signatures in breast cancer serum samples using high-throughput sequencing, while Younis *et al*. (2025) [72]defined prognostic miRNA–gene expression signatures in TNBC cohorts. These datasets similarly implicated miRNAs such as miR-92a-3p and miR-95–3p in breast cancer biology, supporting their potential relevance across diverse patient populations. Nevertheless, as emphasized in these studies, functional and clinical validation is essential before advancing these miRNAs as biomarkers or therapeutic targets.

This study has several important limitations. First, although associations were identified between HTR2A SNPs and miRNA expression, functional validation of these relationships was not performed. In particular, the effects of variants such as rs7322347 on miRNA binding and post-transcriptional regulation remain unverified and require experimental confirmation using approaches such as luciferase reporter assays or CRISPR-based genome editing. Second, functional analyses were limited to a single TNBC cell line (MDA-MB-468), which may restrict generalizability. Validation in additional TNBC models with distinct HTR2A haplotypes, as well as in non-tumorigenic controls, is necessary to confirm reproducibility and genotype-specific effects. Third, while the sample size was adequate for detecting associations with common variants, the study may be underpowered to robustly evaluate rare haplotypes or subtle SNP–miRNA interactions after multiple testing correction. Furthermore, miRNA expression profiling was conducted using NanoString without independent qPCR validation, and the functional roles of identified miRNAs were not directly tested, limiting mechanistic interpretation. Finally, the study population was restricted to AA women. While this focus is important for addressing disparities, it may limit generalizability, and the potential for population stratification cannot be fully excluded. Replication in larger, multiethnic cohorts and independent datasets will be essential to validate these findings and clarify their broader applicability. Collectively, these limitations highlight the need for comprehensive functional and clinical validation to establish the biological relevance of HTR2A-associated miRNA regulation in TNBC.

## Conclusions

This study identifies HTR2A genetic variation as a potential contributor to breast cancer susceptibility in African American women and highlights its association with dysregulated miRNA networks in TNBC models. These findings support a potential link between serotonergic signaling and post-transcriptional regulation in aggressive breast cancer subtypes. Importantly, this work suggests that genetic variation in HTR2A may influence oncogenic signaling pathways through interactions with miRNA-mediated regulatory mechanisms. While these associations provide insight into the molecular underpinnings of breast cancer disparities, they should be interpreted cautiously pending functional validation. Future research should focus on mechanistic studies to validate SNP-dependent effects on miRNA binding and gene regulation, including the use of luciferase reporter assays and CRISPR-based models. Additionally, larger multi-center and multiethnic cohort studies are needed to confirm these findings and assess their generalizability. Integrating genomic, transcriptomic, and functional analyses will be critical to determining the clinical relevance of HTR2A-associated miRNA networks in TNBC.

## Figures and Tables

**Table 1.** Case control study population.

Study Population	
BCa Cases	192
Population Controls	225
TOTAL (cases and controls)	417

**Table 2.** Populations included from the 1000 Genomes Project used for allele frequency comparisons of HTR2A SNPs. African (AFR) and European (EUR) superpopulations and their corresponding subpopulations are shown.

Population	Population Abbreviation	Counts
Super population: AFR	AFR	661
African Caribbean in Barbados	ACB	96
African Ancestry in Southwest US	ASW	61
Esan in Nigeria	ESN	99
Gambian in Western Division, The Gambia	GWD	113
Luhya in Webuye, Kenya	LWK	99
Mende in Sierra Leone	MSL	85
Yoruba in Ibadan, Nigeria	YRI	108
Super population	EUR	503
Utah residents with Northern and Western European ancestry	CEU	99
Finnish in Finland	FIN	99
British in England and Scotland	GBR	91
Iberian populations in Spain	IBS	107
Toscani in Italy	TSI	107

**Table 3.** Characteristics of the selected SNPs in the 5HT2A receptor gene, their location, and the code sequences used to identify their genotypes.

SNP	Location	Catalog No.	Assay ID	Polymorphism Type
Rs6304	Exon 3	4351379	C__11696901_20	Missense Mutation (His452Tyr)
Rs6311	Promoter	4351379	C___8695278_10	−1438G>A
Rs1745837	Intron 1	4351379	C___2711069_20	Transversion substitution
Rs 7322347	Intron 2	4351379	C__29706403_10	Transversion substitution

**Table 4.** Minor allele frequencies of HTR2A SNPs across populations (1000 Genomes Project). Allele frequency data were obtained from the 1000 Genomes Project database and were used for comparative purposes. These data were not generated in this study but serve as a reference for population-level variation in HTR2A SNPs.

SNP	Allele	Minor allele	MAF AFR	MAF EUR	Difference	p-value
rs6304	T/C	C	7.56%	0.00%	7.56%	p < 0.00001
rs6311	C/T	T	40.92%	43.74%	− 2.82%	p = 0.335
rs1745837	C/T	T	99.17%	68.99%	30.18%	p < 0.00001
rs7322347	A/T	T	67.55%	43.54%	24.01%	p < 0.00001

Data source: 1000 Genomes Project (https://www.internationalgenome.org

**Table 5.** Functional significance of HTR2A SNPs based on in silico prediction. CADD scores, regulatory potential, and conservation metrics were used to evaluate the functional relevance of selected HTR2A variants. Higher CADD scores indicate an increased likelihood of deleterious effects, while regulatory and conservation scores provide insight into potential gene regulation and evolutionary importance. These predictions support the potential biological relevance of selected SNPs.

SNP	Allele	Genomic Location	Functional Annotation	CADD Score	Regulatory Potential	Conservation Score	Functional Interpretation
rs6304	T/C	Exon 3 (missense)	nsSNP	11.60	0.382	0.997	Likely affects protein structure and receptor stability
rs6311	C/T	Intron (near promoter region)	*TFBS	10.58	0.450	0.000	May influence transcriptional regulation
rs1745837	C/T	Intron (near gene)	—	5.57	0.000	0.000	Limited predicted functional impact
rs7322347	A/T	Intron	miRNA binding site	3.84	0.054	0.000	May affect miRNA-mediated regulation

CADD (Combined Annotation Dependent Depletion) scores estimate the deleteriousness of genetic variants; scores ≥10 suggest potential functional impact.

* TFBS = transcription factor binding site; nsSNP = non-synonymous SNP.

**Table 6.** Association between HTR2A SNPs and breast cancer risk in AA women.

Model	Genotype	Patients N (%)	Controls N (%)	OR (95% CI)	*p*-Value	*p*_*c*_-Value
**rs6304**						
Codominant	T/T	108 (62.1%)	39 (20.4%)	1.00	**<0.0001**	**<0.0004**
	C/T	64 (36.8%)	13 (6.8%)	1.78 (0.88–3.58)		
	C/C	2 (1.1%)	139 (72.8%)	**0.01 (0.00–0.02)**		
Dominant	T/T	108 (62.1%)	39 (20.4%)	1.00	**<0.0001**	**<0.0004**
	C/T-C/C	66 (37.9%)	152 (79.6%)	**0.16 (0.10–0.25)**		
Recessive	T/T-C/T	172 (98.8%)	52 (27.2%)	1.00	**<0.0001**	**<0.0004**
	C/C	2 (1.1%)	139 (72.8%)	**0.00 (0.00–0.02)**		
Overdominant	T/T-C/C	110 (63.2%)	178 (93.2%)	1.00	**<0.0001**	**<0.0004**
	C/T	64 (36.8%)	13 (6.8%)	**7.97 (4.19–15.14)**		
Log-additive	---	---	---	0.16 (0.12–0.25)	**<0.0001**	**<0.0004**
**rs6311**						
Codominant	T/T	16 (10.1%)	108 (57.5%)	1.00	**<0.0001**	**<0.0004**
Codominant	C/T	80 (50.6%)	44 (23.4%)	**12.27 (6.46–23.30)**	**<0.0001**	**<0.0004**
Dominant	C/C	62 (39.2%)	36 (19.1%)	**11.62 (5.97–22.64)**	**<0.0001**	**<0.0004**
	T/T	16 (10.1%)	108 (57.5%)	1.00		
Dominant	C/T-C/C	142 (89.9%)	80 (42.5%)	**11.98 (6.63–21.66)**	**<0.0001**	**<0.0004**
Recessive	T/T-C/T	96 (60.8%)	152 (80.8%)	1.00	**<0.0001**	**<0.0004**
Recessive	C/C	62 (39.2%)	36 (19.1%)	**2.73 (1.68–4.42)**	**<0.0001**	**<0.0004**
Overdominant	T/T-C/C	78 (49.4%)	144 (76.6%)	1.00	**<0.0001**	**<0.0004**
Overdominant	C/T	80 (50.6%)	44 (23.4%)	**3.36 (2.12–5.32)**	**<0.0001**	**<0.0004**
Log-additive	---	---	---	**3.24 (2.37–4.42)**	<0.0001	**<0.0004**
**rs1745837**						
Codominant	C/C	102 (57%)	162 (90.5%)	1.00	**<0.0001**	**<0.0004**
Codominant	T/C	77 (43%)	16 (8.9%)	**7.64 (4.23–13.83)**	**<0.0001**	**<0.0004**
Dominant	T/T	0 (0%)	1 (0.6%)	0.00 (0.00-NA)	**<0.0001**	**<0.0004**
	C/C	102 (57%)	162 (90.5%)	1.00		
Dominant	T/C-T/T	77 (43%)	17 (9.5%)	**7.19 (4.02–12.86)**	**<0.0001**	**<0.0004**
Recessive	C/C-T/C	179 (100%)	178 (99.4%)	1.00	0.24	0.96
Recessive	T/T	0 (0%)	1 (0.6%)	0.00 (0.00-NA)	0.24	0.96
Overdominant	C/C-T/T	102 (57%)	163 (91.1%)	1.00	**<0.0001**	**<0.0004**
Overdominant	T/C	77 (43%)	16 (8.9%)	**7.69 (4.25–13.91)**	**<0.0001**	**<0.0004**
Log-additive	---	---	-	**6.55 (3.71–11.57)**	<0.0001	**<0.0004**
**rs7322347**						
Codominant	A/A	71 (40.1%)	90 (46.6%)	1.00	0.45	1.8
Codominant	A/T	75 (42.4%)	73 (37.8%)	1.30 (0.83–2.04)	0.45	1.8
Dominant	T/T	31 (17.5%)	30 (15.5%)	1.31 (0.73–2.36)	0.21	0.84
	A/A	71 (40.1%)	90 (46.6%)	1.00		
Dominant	A/T-T/T	106 (59.9%)	103 (53.4%)	1.30 (0.86–1.97)	0.21	0.84
Recessive	A/A-A/T	146 (82.5%)	163 (84.5%)	1.00	0.61	2.44
Recessive	T/T	31 (17.5%)	30 (15.5%)	1.15 (0.67–2.00)	0.61	2.44
Overdominant	A/A-T/T	102 (57.6%)	120 (62.2%)	1.00	0.37	1.48
Overdominant	A/T	75 (42.4%)	73 (37.8%)	1.21 (0.80–1.83)	0.37	1.48
Log-additive	---	---	---	1.18 (0.89–1.56)	0.26	1.04

**Table 7.** Haplotype analysis of HTR2A SNPs and association with breast cancer risk. Haplotypes were constructed based on combinations of four HTR2A SNPs. Frequencies were compared between cases and controls, and associations were evaluated using logistic regression. Haplotypes with low frequency may yield inflated odds ratios and should be interpreted cautiously.

Haplotype	rs6304	rs6311	rs1745837	rs7322347	Frequency	OR(95% CI)	*p*-Value	*p*_*c*_-Value
1	C	T	C	A	0.2688	1.00	---	0.00
2	T	C	C	A	0.1947	**8.11 (4.18 – 15.72)**	<0.0001	<0.0004
3	C	T	C	T	0.1423	1.05 (0.34 – 3.19)	0.93	3.72
4	T	C	C	T	0.0721	**8.46 (3.57 – 20.07)**	<0.0001	<0.0004
5	T	T	C	A	0.071	**6.57 (2.74 – 15.77)**	<0.0001	<0.0004
6	T	C	T	T	0.0697	**193.57 (35.07 – 1068.40)**	<0.0001	<0.0004
7	C	C	C	A	0.0504	0.31 (0.02 – 4.35)	0.39	1.56
8	T	C	T	A	0.0431	**49.11 (7.11 – 338.98)**	1e-04	4e-04
9	T	T	C	T	0.0408	**14.25 (3.58 – 56.75)**	2e-04	8e-04

**Table 8.** Structural organization of the HTR2A protein. The HTR2A receptor is a G protein–coupled receptor (GPCR) characterized by seven transmembrane helices (TM1–TM7), interconnected by extracellular (ECL) and intracellular loops (ICL). The N-terminus is located extracellularly, while the C-terminus is cytoplasmic and involved in intracellular signaling and protein interactions.

Position(s)	Feature key	Domain Location	Structure
1–75	Topological domain	N-terminus	Extracellular
76–99	Transmembrane	TM1	Helical
100–110	Topological domain	ICL1	Cytoplasmic
111–132	Transmembrane	TM2	Helical
133–148	Topological domain	ECL1	Extracellular
149–171	Transmembrane	TM3	Helical
172–191	Topological domain	ICL2	Cytoplasmic
192–215	Transmembrane	TM4	Helical
216–233	Topological domain	ECL2	Extracellular
234–254	Transmembrane	TM5	Helical
255–324	Topological domain	ICL3	Cytoplasmic
325–346	Transmembrane	TM6	Helical
347–362	Topological domain	ELC3	Extracellular
363–384	Transmembrane	TM7	Helical
385–471	Topological domain	C-terminus	Cytoplasmic

**Table 9.** Functional motifs in the HTR2A receptor. Key conserved motifs within the HTR2A protein are essential for receptor activation, signal transduction, and intracellular interactions. These motifs are characteristic of GPCRs and contribute to ligand-induced conformational changes and downstream signaling.

Motif	Residue Position	Structural Location	Functional Role
DRY	172–174	Intracellular loop 2 (ICL2)	Critical for G-protein coupling and receptor activation; regulates signal transduction
NPxxY	376–380	Transmembrane helix 7 (TM7)	Involved in receptor conformational changes and stabilization during activation
PDZ-binding motif	469–471	C-terminal domain	Mediates protein–protein interactions and receptor trafficking

**Table 10.** Predicted impact of rs6304 on HTR2A protein stability. Computational analyses were performed using multiple *in silico* tools to evaluate the thermodynamic and structural effects of the rs6304 (Ile197Val) mutation. Negative ΔΔG values indicate decreased protein stability, whereas positive ΔΔSVib values suggest increased molecular flexibility. All models consistently predict a destabilizing effect of the variant, accompanied by increased structural flexibility.

Method	ΔΔG (kcal/mol)	Predicted Effect on Stability	Functional Interpretation
DynaMut	−0.76	Destabilizing	Reduced protein stability
mCSM	−0.83	Destabilizing	Reduced structural integrity
SDM	−0.18	Destabilizing	Mild destabilizing effect
DUET	−0.51	Destabilizing	Consistent destabilization prediction
ENCoM (ΔΔSVib)	+0.125 (kcal/mol·K)	Increased flexibility	Enhanced molecular motion

**Table 11.** Genotypic profiles of HTR2A SNPs in breast cell lines. Genotype distribution of selected HTR2A SNPs in the non-tumorigenic mammary epithelial cell line (MCF-10A) and the TNBC cell line (MDA-MB-468). The TNBC cell line carries genotypic features consistent with increased breast cancer risk, including the TCCT haplotype and homozygosity for the rs7322347 minor allele, which has been associated with miRNA binding.

SNP	Minor Allele	MCF-10A (Normal)	MDA-MB-468 (TNBC)	Interpretation
rs6304	C	C/T	C/T	Both cell lines heterozygous
rs6311	T	T/T	C/C	Differential genotype between normal and TNBC
rs1745837	T	C/C	C/C	No variant allele detected in either cell line
rs7322347	T	A/T	T/T	TNBC line carries a homozygous variant associated with miRNA binding

**Table 12.** Differentially expressed miRNAs and their functional roles in TNBC. Six miRNAs were consistently upregulated across multiple comparisons in TNBC cells following HTR2A activation. These miRNAs predominantly target tumor suppressor genes and are associated with key oncogenic pathways, including PI3K/AKT signaling, cell cycle regulation, and inflammatory signaling.

miRNA	Expression Pattern*	Key Target(s)	Classification	Functional Role
miR-1270	Upregulated	MAPK10	Oncogenic	Promotes proliferation and migration via MAPK pathway
miR-3147	Upregulated	STAT1	Oncogenic	Suppresses tumor suppressor signaling and promotes cell survival
miR-346	Upregulated	IL-18 (indirect)	Oncogenic	Modulates immune signaling and inflammatory response
miR-377–3p	Upregulated	CDK6, CCND1	Oncogenic	Promotes cell cycle progression and NF-κB pathway activation
miR-92a-3p	Upregulated	PTEN	Oncogenic	Activates PI3K/AKT signaling and promotes proliferation
miR-95–3p	Upregulated	CDK6,CDKN1A (p21)	Oncogenic	Enhances tumorigenesis by promoting G1/S transition

* Expression pattern reflects consistent differential expression across comparisons (treated vs. untreated TNBC, TNBC vs. normal). Statistical significance was defined as p < 0.05.
